# Evaluation of the effectiveness of wearing compression stockings for prevention of occupational edema in hairdressers

**DOI:** 10.1590/1677-5449.190028

**Published:** 2020-03-06

**Authors:** Claudia Guimarães Agle, Cloud Kennedy Couto de Sá, Dejean Sampaio Amorim, Marcondes Antonio de Medeiros Figueiredo

**Affiliations:** 1 Faculdade de Tecnologia e Ciências – FTC, Departamento de Medicina, Salvador, BA, Brasil.; 2 Hospital Geral Ernesto Simões Filho – HGESF, Departamento de Ortopedia, Salvador, BA, Brasil.; 3 Clínica CEAVE, Departamento de Cirurgia Vascular e Endovascular, Salvador, BA, Brasil.; 4 Clínica de Angiologia Dr Marcondes Figueiredo, Departamento de Angiologia, Uberlândia, BA, Brasil.

**Keywords:** compression stockings, occupational health, edema, lower limbs, quality of life

## Abstract

**Background:**

Occupational lower limb edema is an important factor in deterioration of quality of life. Prevention involves prescription of prophylactic measures, such as wearing compression stockings.

**Objectives:**

To evaluate the effectiveness of compression stocking for prevention of occupational edema and its repercussions for the quality of life of hairdressers.

**Methods:**

A clinical trial involving measurements of the ankles (point B) and calves (Point C) of 38 hairdressers without venous disease at the beginning and end of workdays spent wearing or not wearing compression stockings. Participants also answered a questionnaire about symptoms and quality of life in venous disease.

**Results:**

Point B measurements were: 21.1 ± 2.2 cm in the morning without stockings; 22.1 ± 2.3 cm at the end of the day without stockings (p = 0.0001 compared to baseline without stockings); and 21.2 ± 2.1 cm at the end of the day wearing compression stockings (p = 0.0001 compared to the end of day not wearing compression stockings). The comparison between point B values for the start of the day without compression stockings and the end of the day with stockings (p = 0.324) was not significant, showing that there was no lower limb edema at the end of the working day when compression stockings were worn. Improvements were observed in ratings for limitations of work activities (p = 0.0001), domestic activities (p = 0.008) and leisure or social activities performed standing up(p = 0.0001).

**Conclusions:**

Compression stockings are effective for preventing occupational lower limb edema and the attenuation of symptoms such as pain and fatigue directly contributes to better quality of life for hairdressers.

## INTRODUCTION

Many different professions require people to remain standing for long periods in order to perform their jobs, whether standing still or walking. This unnatural position can cause venous stasis and consequent increase in the volume of the lower limbs (LL) as the working day progresses. Edema at the end of the day is a common complaint and is due to a physiological phenomenon, caused by leakage of fluid from venules. This is because of a gradual increase in venous pressure in the dependent parts of the body, caused by gravity. In the majority of healthy people, this evening edema is asymptomatic and disappears from one day to the next. However, unpleasant subjective feelings of heaviness and tiredness may be reported.

Work-related or occupational edema (OE) is a phenomenon that can even be present in people who have no visible or palpable signs or symptoms of venous insufficiency (CEAP class C0) or only have thread veins and telangiectasias (class C1).^1^

According to Berenguer,^2^ by studying the health and working conditions of occupational groups, working processes can be classified and the profile of illness among workers can be described, so that possible associations between occupation and health can be analyzed.

Lower limb edema is considered an important factor in deterioration of quality of life, because the discomfort, premature tiredness, and feelings of heaviness reduce professional productivity.^1^^,^^3^ Occupational edema has also been associated with another factor: studies have proven that wearing inappropriate footwear significantly interferes with regulation and control of movement of the ankle joint, in addition to causing discomfort and circulatory problems. A study conducted with traffic control workers showed that wearing boots combined with the static posture demanded by the job could rapidly provoke muscle fatigue and strangulation of venous and lymphatic capillaries and even involved risk of formation of thrombi in the superficial and deep systems.^4^ As a result, hemodynamic venous disorders occur in people with no symptoms of any type of vascular problem, but whose professions expose them to working constantly in a standing position.

Prevention of OE involves prescription of prophylactic measures, such as rest periods lying down during the working day, in order to reduce venous pressure.^1^ Although walking and physical exercises in water^5^^,^^6^ helped reduce OE, the best results from preventative measures are achieved by wearing compression stockings (CSs).^7^^-^9 Some groups of workers have worn CSs, but not systematically. Studies designed to assess their efficacy and compliance among target populations are still rare, and a lack of knowledge about the risks of LL edema means that people associate wearing CSs with treatment of specific conditions or refuse to wear them because of climatic conditions or esthetic appearance. In this study, the effect of wearing CSs was assessed among workers with no apparent venous disease, analyzing the relationship with edema accumulated over the course of the working day. The study objective is to evaluate the efficacy of wearing CSs for prevention of OE and its repercussions for the quality of life of hairdressers.

## METHOD

### Study design

Clinical prevention trial (the article is not assessing treatment of a disease, but is designed to document its prevention); single group (there was no control group; the assessments are conducted with the same group at different times); unblinded (both the investigator and the participants knew what intervention was being administered); and single arm (all participants received the same intervention).

The study design was defined according to the standardized classification used by the Brazilian Clinical Trials Register (ReBEC).

### Sample and sample size calculation

Sample size^10^ was estimated at 38 individuals using PEPI for Windows, based on a 5% significance level, 80% statistical power, and an expected difference of 10% in comparisons between leg circumference deltas (the outcome used to calculate sample size) from Assessment 1 to Assessment 2, with a standard deviation of 1.5 times the value of the mean. Volunteers were recruited at random at beauty salons in the city of Salvador, BA, Brazil, and were analyzed in a single study group. Volunteers were enrolled if they met the inclusion criteria, without drawing lots: all were asked whether they would be willing to participate in the study and, after consent had been given, volunteers were only enrolled if they worked for at least 8 hours standing up, with 30 minute intervals, and were classified as CEAP categories C0 or C1,^11^ until a total of 38 had been recruited. Table 1 lists descriptive data on the 38 hairdressers who took part.

**Table 1:** Characteristics of the hairdressers who participated in the study of the effects of wearing elastic stockings on indicators of quality of life and symptoms of venous disease (n = 38).

**Variable**	**Mean ± SD**	**Minimum value**	**Maximum value**
Age (years)	41.7±9.4	25	60
Weight (kg)	66.8±14.0	52.0	96.0
Height (m)	1.61±0.06	1.41	1.70
BMI (kg/m^2^)	25.7±4.9	19.9	37.3

SD: standard deviation; BMI: body mass index.

The following exclusion criteria were adopted: refusal to take part, taking medication that could influence formation of LL edema, and systemic diseases such as heart failure and renal, hepatic, thyroid, or rheumatic disease.

### Procedures

**Figure 1:**
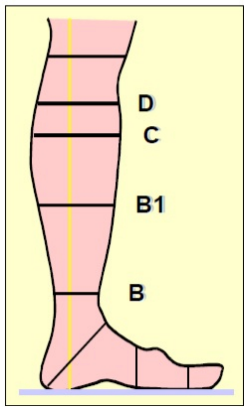
Points where stockings are assessed to define the compression profile, as recommended by the European Committee for Standardization^12^. B: smallest ankle circumference; B1: point at which the Achilles tendon enters the calf; C: largest circumference of the calf; D: point below the tuberosity of the tibia.

Participants’ ankles (point B) and calves (point C) (Figure 1) were measured at the start and end of their work shifts (8 hours standing up). The morning measurements were used as the criterion for choosing the size of the stockings, since point B and point C are the references recommended for choosing the correct size of below-the-knee stockings. When the hairdressers arrived at the beauty salon, before starting work, one of the study authors was waiting for them at the salon and took their point B and point C measurements using a tape measure (in centimeters). The corresponding measurements were taken again at the end of the working day: the author measured points B and C at the appointed time, still in the beauty salon. No time elapsed between ending work and being measured. For the first data collection session (Assessment 1, after 8 hours standing up), participants spent their working day as normal, with no instructions to take prophylactic measures against volumetric changes to the LL. For the second data collection (Assessment 2), they all performed their jobs while wearing below-the-knee CSs with 18 to 20 mmHg compression (Venosan© - Abreu e Lima, Pernambuco, Brazil). The Assessment 2 data collection consisted of taking the same measurements at the end of the day, after wearing the CSs for 7 days. However, this 7-day period began after an 8-day period of adaptation to the CS. Assessment 2 (the repeat measurements) was therefore 15 days after Assessment 1.

It was unnecessary to measure diameters at the start of the working day on which participants were wearing the stockings, since the objective was to determine whether or not there was edema at the end of the day and compare the result with the end of the day not wearing stockings. Morning measurements were only taken at Assessment 1 (without stockings) for comparison, and were recorded. Stockings were put on within half an hour of getting out of bed and were worn until the end of the work shift, when ankle and calf measurements were taken. The volunteers were given instructions on how to wear and take care of the stockings. A comparative questionnaire on symptoms and quality of life in venous disease was administered at the end of both data collection sessions. This was read out loud by the study author to each participant, who chose scores for questions on pain and limitations to daily activities.

All measurements were taken and all questionnaires were administered by the same person (one of the study authors).

Recruitment and follow-up of patients took 6 months (April-October 2018) and an average of three patients were assessed every 15 days. All participants were provided with detailed information on the objectives of the study and the risks and benefits involved in the procedures and signed free and informed consent forms. The research project was submitted for appraisal by the Research Ethics Committee at the Faculdade de Tecnologia e Ciências (FTC), Salvador, BA, Brazil (CAAE 86437418.6.0000.5032) and approved on the Plataforma Brasil. It was also registered and approved by the ReBEC.

### Questionnaires

First, a questionnaire on general data for identification and characterization of the sample was administered. Symptoms and quality of life in venous disease were then assessed using the Brazilian Portuguese version of the VEnous INsufficiency Epidemiological and Economic Study - Quality of Life/Symptoms (VEINES-QOL/Sym) questionnaire.^13^ The questionnaire was administered in an interview format.

The VEINES-QOL/Sym produces two scores, the first, the VEINES-QOL, estimates the impact of chronic venous disease (CVD) on quality of life, and the VEINES-Sym deals with symptoms caused by CVD. The total VEINES-QOL score is calculated using the 25 items that make up questions 1, 3, 4, 5, 6, 7, and 8, while question 2 covers the times of day when symptoms are most intense.

The VEINES-Sym score is based on ten items (questions 1 and 7). Nine of these are related to symptoms: heavy legs, aching legs, swelling, night cramps, heat or burning sensation, restless legs, throbbing, itching, and tingling sensation. These symptoms are rated in terms of frequency on a five-point Likert scale. The final item, question 7, is related to leg pain and is rated for intensity on a six-point Likert scale.^13^

### Statistical analysis

The independent variable was categorized as Assessment 1 (without stockings) and Assessment 2 (with stockings). The primary dependent variables were volunteers’ leg circumference measurements and VEINES-QOL and VEINES-Sym scores at Assessments 1 and 2.

Mean leg circumference measurements at Assessments 1 and 2 were compared using the *t* test for dependent samples, with a significance level of 5%.

The Wilcoxon test was used to compare median VEINES-QOL and VEINES-Sym scores and ratings for heavy, aching, and swollen legs from the VEINES questionnaire from Assessments 1 and 2, with a 5% significance level.

All analyses were conducted using the Statistical Package for the Social Sciences (SPSS) for Windows, version 15.0. Data were expressed as mean ± standard deviation or, when appropriate, as median and interquartile range.

## RESULTS

**Figure 2:**
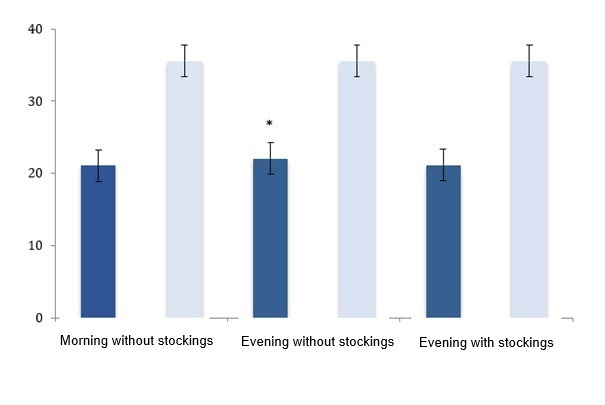
Effect of wearing elastic stockings on formation of occupational edema in hairdressers who work standing up (n = 38). Dark bars: point B; Pale bars: point C. * p = 0.0001 for start of day without stockings against end of day with stockings.

As illustrated in Figure 2, the values for point B were 21.1±2.2 cm at the start of the day without stockings; 22.1±2.3 cm at the end of the day without stockings (p = 0.0001 in relation to the start of the day without stockings); and 21.2±2.1 cm at the end of the day with stockings (p = 0.0001 in relation to the value for the end of the day without stockings). The difference between mean values for point B at the start of the day without stockings and at the end of the day with stockings was not significant (p = 0.324). There were no changes in the point C measurements for any of the study participants at any of the Assessments.

Table 2 lists results showing the significant effect of wearing elastic stockings on quality of life indicators (VEINES-QOL; p = 0.0001) and symptoms (VEINES-Sym; p = 0.0001) of CVD in hairdressers who work standing up. Table 2 also shows results for the comparisons of symptoms of heavy, aching, and swollen legs, with and without stockings.

**Table 2:** Effects of wearing elastic stockings on indicators of quality of life and symptoms of chronic venous disease in hairdressers who work standing up (n = 38).

**Score**	**Without stockings**	**With stockings**	***p*-value** ^*^
	**Median (IQR)**	**Median (IQR)**	
VEINES-Sym	32 (27-38)	51 (47-51)	0.0001
VEINES-QOL	81 (68-86)	103 (94-106)	0.0001
Heavy legs	2 (1-2)	5 (5-5)	0.0001
Aching legs	2 (1-3)	5 (5-5)	0.0001
Swollen Legs	2.5 (2-5)	5 (5-5)	0.0001

IQR: interquartile range; VEINES-QOL/Sym: Venous Insufficiency Epidemiological and Economic Study – Quality of life/Symptoms;

* according to the Wilcoxon test.

Table 3 lists the results of statistical tests conducted to detect a possible effect of wearing stockings on limitations to typical employment, domestic, and social or leisure activities in hairdressers who work standing up. It can be observed that there were improvements in limitations affecting employment activities (p = 0.0001), domestic activities (p = 0.008), and leisure or social activities performed standing up (p = 0.0001), but no change in limitations affecting social or leisure activities when sitting (p = 0.0317).

**Table 3:** Effects of wearing elastic stockings on limitations to typical employment, domestic, and social or leisure activities in hairdressers who work standing up (n = 38).

**Activities**	**Without stockings**	**With stockings**	***p*-value** ^*^
	**Median (IQR)**	**Median (IQR)**	
Employment	2 (2-3)	3 (3-3)	0.0001
Domestic	3 (3-3)	3 (3-3)	0.008
Social or leisure, standing	3 (2-3)	3 (3-3)	0.0001
Social or leisure, sitting	3 (3-3)	3 (3-3)	0.317

IQR: interquartile range; Questions are taken from the Venous Insufficiency Epidemiological and Economic Study – Quality of life (VEINES-QOL) questionnaire . Higher values indicate less limitation.

* according to the Wilcoxon test.

## DISCUSSION

It is known that variations in venous hemodynamics over the course of the day are caused by separation of the cusps of the valves, with a consequent increase in venous reflux.^14^ Venous return is a process that has to overcome the force of gravity and involves a range of different compensating mechanisms, including the impulse-aspiration pumps (IAPs), described by Brizzio in Argentina during the 1980s. According to Godoy,^15^ if a human being remains standing up and immobile, these pumps do not function and this failure is a cause associated with occupational edema.^16^ In the case of people who remain in a sedentary position all day long, it appears that there is a reduction in musculoarticular work, which makes venous stasis more likely.

Bishara et al.^17^ suggest that this change in venous hemodynamics as the working day progresses may be a consequence of disorders of valve competence, which is essential for normal venous function. They observed a significant reduction in venous capacitance measured by photoplethysmography during the afternoon, in comparison with the morning, and attributed this difference to the large volume of blood contained in the veins of the lower limbs of the study participants after they had spent a long time standing up.

In a previous study, we observed that nurses with no signs of chronic venous insufficiency who work standing up for 90% of their work shifts had high venous pressure levels in their LL and overproduction of reactive oxygen species after work. These free radicals are mediators of vessel wall damage, and oxidative damage to the endothelial membrane increases vascular permeability, with consequent edema.^18^^,^^19^

According to Godoy,^15^ 2/3 of the conditions that affect the circulatory return system can be controlled by the compression method alone. Although it is underestimated by many health professionals, its efficacy has been proven since ancient times, when Hebrews and Greeks used compressive dressings to treat ulcers and Roman soldiers bound their legs to better withstand long marches during the wars. Today, with these conditions becoming ever more common, the high incidence of venous and lymphatic disease is attributed to the modern lifestyle, rooted in industrialization and computerization, which oblige a majority of people to remain standing up or in other harmful positions for long periods. It has been shown that indigenous people with no signs of these illnesses begin to exhibit them when they move to large urban centers.^15^

The primary objective of compression therapy is to rebalance tissues and interstitium, exerting an external pressure that can counteract the pathological intravascular and interstitial internal pressures. The most common indication for compression therapy is edema.^1^^,^^15^ It even acts on the mediators involved in localized inflammatory reactions at the micorcirculatory level, which may explain the relief that is felt when adequately administrated. The increased micorcirculatory velocity can be demonstrated by laser-Doppler flowmetry. Godoy et al.^15^ showed that compression increased cutaneous oxygenation during venous insufficiency. Partsch et al. also demonstrated reduction in venous reflux, even in segments without valves.^20^

In this study, we were able to confirm a considerable increase in the volume of both LL in the evening, after an 8 hour working day when not using any type of prophylactic measure against OE. With regard to patients who did not have significant edema measured at the end of the day, it is possible that there was formation of subclinical edema that was not diagnosed by means of circumference measurements. This could explain the complaints of swollen and heavy legs at the end of the day. It could also be an indication of study bias, since circumference measurements in centimeters are not as faithful or sensitive to small changes as water plethysmography, for example. Other studies designed to capture this aspect are needed to confirm this hypothesis.

Although Figure 2 illustrates a small difference in leg circumference (just 1 cm difference between mean point B measurements), it can be inferred that these statistically significant values were also clinically significant, since, while there is no reference in the literature that defines an exact diameter at which edema is considered important, the correlation of the symptoms reported by the patients was more intense and more frequent the larger the circumference measured.

The fact is that OE is uncomfortable, and the sensations of heaviness and tiredness documented in our results can lead to reduced productivity, with increased absenteeism from work and poor quality of life, and may be one of the first manifestations of decompensation of the venous and lymphatic systems,^21^ in particular in individuals with greater body mass index.^22^

The majority of study participants did not only report a daytime limitation at work because of leg problems, but also limitations related to daily activities at home (housework, routine tasks, gardening, etc.). The same was true of social or leisure activities that involve remaining standing for long periods (parties, weddings, public transport, etc.). The improvement observed when wearing CSs confirms their effectiveness for prevention of OE and their direct impact on the quality of life of the hairdressers.

In order to correctly prescribe CSs, whether for therapeutic or prophylactic purposes, it is essential that the physician is familiar with normal pressure values and with the pressure values caused by specific conditions, to be able to choose a compression level that will combat venous stasis, whether under physiological or pathological conditions. According to Partsch et al.,^20^ the optimal pressure for reducing edema of the extremities is still under debate. Since the participants in this study did not have any obvious clinical manifestation of venous disease, we chose elastic stockings with a pressure level of 18-20 mmHg, which proved to be safe and effective, and higher compression was unnecessary. Belczak et al. concluded in a recent study that compression of 20-30 mmHg is more effective for people who work sitting down, but is not so significant for those who work standing up. For these people, a level of 15-20 mmHg achieved good results and benefits were reported from 10 mmHg.^23^

Thus, in addition to specifying the pressure necessary in mmHg (universal measure), the physician prescribing CSs should also define the model of hosiery to be worn (calf, thigh, panty-hose, or unilateral), specifying the brand, the period to wear them, and the technique for putting them on. Calf-length stockings improve venous hemodynamics and are generally the most appropriate because, in addition to being easy to put on and having better patient compliance, a large proportion of venous and lymphatic problems develop in the lower third of the legs. Additionally, stockings made from good quality materials and technically correctly can last up to 6 months, which makes them an accessible and economic option from the therapeutic and prophylactic perspectives.^1^

One limitation of this study was not conducting Doppler scans, since patients who are apparently free from venous disease may have some other type of disorder that is not detected by physical examination. Additionally, although there are no reports in the literature associating OE with the menstrual cycle, this is also a factor that merits investigation. It is known that the premenstrual period is associated with changes to electrolyte and water metabolism, with physiological retention of extracellular fluid (edema), resulting in hyperestrogenemia.^24^ In our study, it was not possible to correlate the menstrual cycle with increased variation in LL edema, because a large proportion of the patients analyzed were taking contraceptive pills.

The placebo effect may be considered a source of bias in this study. Wearing CSs may, to a certain extent, alter the subjective responses to the questionnaire on pain. However, we consider that this bias would only be applicable to patients whose circumference measurements were unchanged at the end of the working day. For those patients whose significant edema was reduced by wearing CSs, there was a physiological correlation with improved symptoms, which validates the effect of the stockings on OE and its effects.

Another aspect that should be mentioned as a limitation to this study was the great difficulty encountered in finding patients willing to participate in the study. This factor also prevented us from increasing the size of the sample. Wearing CSs is still seen as bothersome and esthetically undesirable, which makes compliance with treatment less likely. This is a problem that must be investigated, since the outcome of treatment is completely dependent on correct and continuous use of the stockings.

On the basis of our findings and those of other researchers already cited, we therefore recommend wearing CSs as a prophylactic measure against OE and its consequences. The absence of statistical significance indicates that there was no difference in the circumference measurements (edema) taken at the start of the day in the morning and at the end of the day wearing stockings, since all of the other comparisons between wearing stockings and not wearing stockings were significant and exhibited variations measured in centimeters. It can therefore be stated that CSs prevented OE.

Beyond simply confirming previous studies that found evidence of the value of wearing CSs as an effective measure for prevention of OE,^19^^,^^25^^,^^26^ the fact that we observed significant results with a heterogeneous sample, in which participants varied in terms of weight, height, and age, should be seen as a positive factor, since it is evidence of the effectiveness of CSs for prevention of OE.

Since venous filling time is primarily determined by venous valve competence, the results of this study may indicate that a certain degree of venous valve incompetence tends to develop in normal lower limbs after prolonged activity in an erect position. A relatively higher degree of venous valve incompetence may be the physiological basis that explains development of pain or swelling in some people’s lower limbs after these activities.

Compression stockings are, therefore, an effective and economic tool for prevention of OE and its long term consequences, and health professionals should encourage wearing them. Their sale by responsible firms should also be increased, since light compression stockings are under publicized, but can be sold without medical prescription and are ideal for this type of edema.
