# Supplementary material for: Evaluation of the effectiveness of wearing compression stockings for prevention of occupational edema in hairdressers
Source: J Vasc Bras. 2020 Mar 6;19:e20190028. doi: 10.1590/1677-5449.190028 (PMC8205113; doi:10.1590/1677-5449.190028)
Supplement: Translation

# Translation: Avaliação da efetividade do uso de meias de compressão na prevenção do edema ocupacional em cabeleireiras

**Authors:** Claudia Guimarães Agle, Cloud Kennedy Couto de Sá, Dejean Sampaio Amorim, Marcondes Antonio de Medeiros Figueiredo

**Affiliations:** 1 Faculdade de Tecnologia e Ciências – FTC, Departamento de Medicina, Salvador, BA, Brasil.; 2 Hospital Geral Ernesto Simões Filho – HGESF, Departamento de Ortopedia, Salvador, BA, Brasil.; 3 Clínica CEAVE, Departamento de Cirurgia Vascular e Endovascular, Salvador, BA, Brasil.; 4 Clínica de Angiologia Dr Marcondes Figueiredo, Departamento de Angiologia, Uberlândia, BA, Brasil.

**Keywords:** meias de compressão, saúde ocupacional, edema, membros inferiores, qualidade de vida

## Abstract

**Contexto:**

O edema ocupacional (EO) de membros inferiores (MMII) é um importante fator de queda na qualidade de vida, e a sua prevenção impõe a prescrição de medidas profiláticas, como o uso de meias de compressão (MCs).

**Objetivos:**

Avaliar a efetividade das MCs na prevenção do EO e a sua repercussão na qualidade de vida de cabeleireiras.

**Métodos:**

Este ensaio clínico realizou medidas de tornozelo e panturrilha de 38 cabeleireiras sem doença venosa no início e no final da jornada de trabalho em um momento sem e em outro usando MCs. Também responderam um questionário sobre sintomas e qualidade de vida em doença venosa.

**Resultados:**

Os valores do ponto B foram de 21,1±2,2 cm no momento inicial sem meias, 22,1±2,3 cm no momento final sem meias (p = 0,0001 em relação ao inicial sem meias), e 21,2±2,1 cm no momento final com meias (p = 0,0001 em relação ao final sem meias). Não foi significante a diferença entre os valores médios do ponto B inicial sem meias e final com meias (p=0,324), ou seja, não houve formação de edema nos MMII ao final da jornada de trabalho em ortostatismo prolongado quando em uso de MCs. Pôde-se observar melhora da limitação sobre as atividades laborais (p = 0,0001), domésticas (p = 0,008) e de lazer ou sociais em pé (p = 0,0001).

**Conclusões:**

As MCs são efetivas na prevenção do EO de MMII, e a atenuação de sintomas como dor e fadiga contribui diretamente para melhor qualidade de vida de cabeleireiras.

## INTRODUÇÃO

Muitas profissões requerem que o indivíduo, no exercício de suas funções, mantenha-se por longos períodos em ortostatismo, seja exclusivamente na posição em pé ou andando. Essa situação antinatural pode resultar em estase venosa e consequente aumento de volume dos membros inferiores (MMII) ao longo da jornada diária de trabalho. O edema comumente relatado ao final de um dia de atividade laboral é resultado de um fenômeno fisiológico, causado pelo extravasamento de fluido das vênulas. Isso ocorre devido a um gradual aumento da pressão venosa nas regiões dependentes do corpo, por conta da gravidade. Em pessoas saudáveis, esse edema vespertino é, em sua maioria, assintomático e desaparecerá de um dia para outro. No entanto, percepções subjetivas desagradáveis de peso e cansaço podem ser reportadas.

Define-se como edema laboral ou ocupacional (EO) o fenômeno que ocorre mesmo em indivíduos sem quaisquer sinais ou sintomas visíveis ou palpáveis de insuficiência venosa (classe C0 na classificação CEAP), ou naqueles somente com microvarizes e telangiectasias (classe C1)^1^.

Segundo Berenguer^2^, o estudo das condições de saúde e trabalho de grupos ocupacionais permite caracterizar os processos laborais e descrever o perfil de adoecimento dos trabalhadores, avaliando possíveis associações entre ocupação e saúde.

O edema de MMII é considerado importante fator de queda na qualidade de vida, pois o desconforto, o cansaço precoce e a sensação de peso diminuem o rendimento profissional^1^^,^^3^. O EO também foi associado a outro fator: estudos comprovaram que o uso de calçado inapropriado interfere significativamente na regulação e no controle de movimento da articulação do tornozelo, além de causar desconforto e problemas circulatórios. Em pesquisa realizada com agentes de trânsito, mostrou-se que o uso de coturnos, somado ao trabalho estático exigido pela postura, pode provocar rapidamente fadiga muscular e o estrangulamento dos capilares venosos e linfáticos, trazendo inclusive o risco de formação de trombos nos sistemas superficial e profundo^4^. Dessa forma, alterações hemodinâmicas venosas em indivíduos sem sintomas de qualquer alteração vascular têm ocorrido entre os profissionais expostos ao trabalho constante em bipedestação.

A prevenção do EO impõe a prescrição de medidas profiláticas, como intervalos de repouso em decúbito no meio do período laboral visando reduzir a pressão venosa^1^. Apesar de andar e praticar exercícios físicos na água^5^^,^^6^ ajudarem a reduzir o EO, as melhores medidas preventivas são conseguidas com o uso de meias de compressão (MCs)^7^^-^9. As MCs têm sido utilizadas por alguns grupos de trabalhadores, mas não sistematicamente. Estudos que se dedicam a avaliar a sua eficiência e aderência pela população ainda são escassos, e a falta de conhecimento dos riscos relacionados ao edema de MMII leva a população a associar o uso de MCs a condições específicas de tratamento ou a rejeitar a sua utilização com base na condição climática e em aspectos estéticos. Neste estudo, foi avaliado o uso de MCs em profissionais sem doença venosa aparente, e a sua relação com a presença de edema acumulado ao longo da jornada de trabalho.O objetivo do presente estudo foi avaliar a efetividade do uso de MCs na prevenção do EO e a sua repercussão na qualidade de vida de cabeleireiras.

## MÉTODO

### Desenho do estudo

Ensaio clínico de prevenção (o artigo não visa tratar uma doença e sim constatar a sua prevenção); grupo único (não houve grupo controle, a avaliação foi realizada em um mesmo grupo em momentos distintos); aberto (tanto o investigador como o participante sabem qual intervenção está sendo administrada); braço único (todos os participantes têm a mesma intervenção).

O delineamento do tipo de estudo foi descrito de acordo com a classificação padronizada pelo Registro Brasileiro de Ensaios Clínicos (ReBEC).

### Amostra e cálculo amostral

O tamanho amostral^10^ foi estimado pelo PEPI for Windows em 38 indivíduos, tendo como referência um nível de significância de 5%, um poder estatístico de 80% e uma diferença esperada de 10% na comparação dos deltas das circunferências de perna (desfecho utilizado para calcular o tamanho da amostra) entre os Momentos 1 e 2, com desvio padrão de 1,5 vezes o valor da média. As voluntárias foram recrutadas aleatoriamente em salões de beleza em Salvador (BA) e compuseram um único grupo. Essa convocação atendeu aos critérios de inclusão, sem sorteio: todas foram questionadas a respeito do interesse em participar do estudo e, após aceitarem, foram incluídas apenas as participantes que trabalhavam no mínimo 8 horas em ortostatismo, com 30 minutos de intervalo, e que se encaixavam nas categorias C0 ou C1 da classificação CEAP^11^, até serem preenchidas as 38 vagas. A Tabela 1 fornece os dados descritivos das 38 cabeleireiras participantes.

**Tabela 1:** Características das cabeleireiras participantes do estudo sobre o efeito do uso de meias elásticas sobre indicadores de qualidade de vida e sintomas de doença venosa (n = 38).

**Variável**	**Média ± DP**	**Valor mínimo**	**Valor máximo**
Idade (anos)	41,7±9,4	25	60
Peso (kg)	66,8±14,0	52,0	96,0
Estatura (m)	1,61±0,06	1,41	1,70
IMC (kg/m^2^)	25,7±4,9	19,9	37,3

DP: desvio padrão; IMC: índice de massa corporal.

Foram adotados como critérios de exclusão: discordar em participar do estudo, estar em uso de medicação que pudesse influenciar na formação de edema dos MMII, ou apresentar doenças sistêmicas como insuficiência cardíaca, renal, hepática, tireoidiana ou reumática.

### Procedimentos

Os indivíduos foram submetidos a medidas de tornozelo (ponto B) e panturrilha (ponto C) (Figura 1) ao início e ao término do período laboral (8 horas em bipedestação). As medidas matinais foram o critério usado para escolher o tamanho das meias, já que o ponto B e o ponto C são a referência indicada para escolha do tamanho adequado das meias 3/4. Ao chegar ao salão, antes de começar a trabalhar, as cabelereiras tinham as suas medidas de pontos B e C obtidas utilizando fita métrica (em centímetros) por um dos autores do trabalho, que já as aguardava no local. Da mesma forma ocorreram as medidas correspondentes ao final da jornada laboral: o autor realizava novamente as medidas dos pontos B e C no horário estipulado, ainda dentro do salão de beleza. Não houve intervalo de tempo entre o término do período de trabalho e as medidas realizadas. Na primeira avaliação, que chamamos de Momento 1 (8 horas de ortostatismo), os indivíduos realizaram sua jornada de trabalho normalmente, sem orientação para qualquer medida profilática contra variações volumétricas dos MMII. Já na segunda avaliação, o Momento 2, todos eles realizaram as atividades laborais usando MCs 3/4 com compressão entre 18 a 20 mmHg (Venosan© - Abreu e Lima, Pernambuco, Brasil). Esse Momento 2 consistiu em reavaliar as medidas ao final do dia de trabalho após uso regular das MCs durante 7 dias. Porém, o uso regular só se iniciou após um período de adaptação às MC de 8 dias pelo usuário. Sendo assim, o Momento 2 (reavaliação das medidas) só ocorreu 15 dias após o Momento 1.

**Figura 1:** Pontos onde as meias são avaliadas para definir o perfil de compressão, conforme preconizado pelo European Committe for Standardization^12^. B: circunferência mínima de tornozelo; B1: ponto em que o tendão de Aquiles entra na panturrilha; C: circunferência máxima da panturrilha; D: ponto abaixo da tuberosidade da tíbia.

Não foi necessária a medida dos diâmetros ao início da jornada laboral quando em uso das meias, já que o objetivo era avaliar a formação ou não de edema ao final do dia, comparativamente ao final do dia no momento sem meias. As medidas matinais foram feitas no Momento 1 (sem meias) para comparação, e foram devidamente registradas. As meias foram colocadas na primeira meia hora após levantar da cama, sendo utilizadas até ao final da jornada de trabalho, quando as medidas de tornozelo e panturrilha foram novamente obtidas. As usuárias receberam instruções sobre a forma e os cuidados com o uso das meias. Foi aplicado um questionário comparativo de sintomas e qualidade de vida em doença venosa ao final de ambos os momentos. Este era lido pelo autor em voz alta para cada paciente, que atribuía uma nota correspondente a um escore proporcional à sua dor e às suas limitações diárias.

A verificação das medidas dos diâmetros, assim como a aplicação dos questionários, foram sempre realizadas pela mesma pessoa (um dos autores do trabalho).

O recrutamento e o acompanhamento das pacientes durou 6 meses (abril-outubro de 2018), e foram avaliadas em média três pacientes a cada 15 dias. Todos os sujeitos receberam detalhadamente as informações sobre os objetivos do estudo, os riscos e os benefícios envolvidos nos procedimentos e assinaram consentimento livre e esclarecido. O projeto de pesquisa foi submetido ao crivo do Comitê de Ética em Pesquisa da Faculdade de Tecnologia e Ciências (FTC), Salvador, BA, Brasil (CAAE 86437418.6.0000.5032) e aprovado na Plataforma Brasil. Também foi registrado e aprovado pelo ReBEC.

### Questionários

Inicialmente, as pacientes responderam um questionário de dados gerais apenas para identificação e caracterização da amostra. A avaliação dos sintomas e da qualidade de vida em doença venosa foi realizada por meio do questionário *VEnous INsufficiency Epidemiological and Economic Study (VEINES) - Quality of Life/Symptoms* (VEINES-QOL/Sym), versão em português do Brasil^13^. As pacientes foram orientadas a responder o questionário, administrado na forma de entrevista.

O VEINES-QOL/Sym produz dois escores, sendo um a estimativa do impacto da doença venosa crônica (DVC) na qualidade de vida, o VEINES-QOL, e o outro relacionado aos sintomas decorrentes da DVC, o VEINES-Sym. Para obtenção do escore total do VEINES-QOL, devem ser considerados os 25 itens que compreendem as questões 1, 3, 4, 5, 6, 7 e 8 do questionário, e a questão 2 deve ser relacionada ao horário do dia em que os sintomas são mais intensos.

O escore produzido pelo VEINES-Sym inclui dez itens (questões 1 e 7). Destes, nove estão relacionados aos sintomas: pernas pesadas, pernas doloridas, inchaço, câimbras noturnas, sensação de calor ou queimação, pernas inquietas, latejamento, coceira e sensação de formigamento. Tais sintomas são avaliados de acordo com a frequência, utilizando uma escala Likert de cinco pontos. O outro item, a questão 7, está relacionado à dor nas pernas e é avaliado quanto à intensidade por uma escala Likert de seis pontos^13^.

### Análise estatística

A variável independente foi categorizada em Momento 1 (sem meias) e Momento 2 (com meias). As variáveis dependentes primárias foram as medidas de circunferência da perna das voluntárias e os escores VEINES-QOL e VEINES-Sym nos Momentos 1 e 2.

As médias nos Momentos 1 e 2 das medidas circunferenciais da perna foram comparadas por meio do teste de teste *t* para amostras dependentes, com o nível de significância estabelecido em 5%.

As medianas dos escores dos VEINES-QOL e VEINES-Sym e das questões sobre pernas pesadas, doloridas e inchadas do questionário VEINES nos Momentos 1 e 2 foram comparadas utilizando o teste de Wilcoxon, com nível de significância de 5%.

Todas as análises foram realizadas no Statistical Package for the Social Science (SPSS) for Windows, versão 15.0. Os dados foram apresentados em média ± desvio padrão ou, quando necessário, em mediana e intervalo interquartil.

## RESULTADOS

Conforme observado na Figura 2, os valores do ponto B foi de 21,1±2,2 cm no momento inicial sem meias; 22,1±2,3 cm no momento final sem meias (p = 0,0001 em relação ao inicial sem meias); e 21,2±2,1 cm no momento final com meias (p = 0,0001 em relação ao final sem meias). Não foi significante a diferença entre os valores médios do ponto B inicial sem meias e final com meias (p = 0,324). Já em relação ao ponto C, não houve alteração nas medidas em nenhum sujeito do estudo em nenhum dos momentos.

**Figura 2:** Efeito do uso de meias elásticas sobre a formação de edema ocupacional em cabeleireiras que trabalham em ortostatismo (n = 38). Barras escuras: ponto B; Barras claras: ponto C. * p = 0,0001 em relação ao início sem meias e ao final com meias.

Na Tabela 2, pode ser observado o efeito significante do uso das meias elásticas sobre indicadores da qualidade de vida (VEINES-QOL; p = 0,0001) e sintomas (VEINES-Sym; p = 0,0001) de DCV em cabeleireiras que trabalham em ortostatismo. Conforme Tabela 2, também foram comparados os sintomas pernas pesadas, doloridas e inchadas sem e com uso das meias.

**Tabela 2:** Efeito do uso das meias elásticas sobre indicadores de qualidade de vida e sintomas de doença venosa crônica em cabeleireiras que trabalham em ortostatismo (n = 38).

**Escore**	**Sem meias**	**Com meias**	**p-valor** ^*^
	**Mediana (IIQ)**	**Mediana (IIQ)**	
VEINES-Sym	32 (27-38)	51 (47-51)	0,0001
VEINES-QOL	81 (68-86)	103 (94-106)	0,0001
Pernas pesadas	2 (1-2)	5 (5-5)	0,0001
Pernas doloridas	2 (1-3)	5 (5-5)	0,0001
Pernas inchadas	2,5 (2-5)	5 (5-5)	0,0001

IIQ: intervalo interquartil; VEINES-QOL/Sym: *Venous Insufficiency Epidemiological and Economic Study – Quality of life/Symptom*;

* obtido pelo teste de Wilcoxon.

A Tabela 3 apresenta o resultado de testes realizados para verificar o possível efeito do uso das meias sobre a limitação imposta às típicas atividades laborais, domésticas e sociais ou de lazer em cabeleireiras que trabalham em ortostatismo. Pode-se observar melhora da limitação sobre as atividades laborais (p = 0,0001), domésticas (p = 0,008) e lazer ou sociais em pé (p = 0,0001), enquanto que não houve mudança sobre a limitação às atividades sociais ou de lazer sentadas (p = 0,0317).

**Tabela 3:** Efeito do uso das meias elásticas sobre a limitação imposta às típicas atividades laborais, domésticas e sociais ou de lazer em cabeleireiras que trabalham em ortostatismo (n = 38).

**Atividades**	**Sem meias**	**Com meias**	**p-valor** ^*^
	**Mediana (IIQ)**	**Mediana (IIQ)**	
Laborais	2 (2-3)	3 (3-3)	0,0001
Domésticas	3 (3-3)	3 (3-3)	0,008
Sociais ou de lazer em pé	3 (2-3)	3 (3-3)	0,0001
Sociais ou de lazer sentadas	3 (3-3)	3 (3-3)	0,317

IIQ: intervalo interquartil; Questões representam recorte do questionário *Venous Insufficiency Epidemiological and Economic Study – Quality of life* (VEINES-QOL). Maior valor representa menos o menor limitação;

* obtido pelo teste de Wilcoxon.

## DISCUSSÃO

Sabe-se que variações hemodinâmicas venosas ao longo do dia decorrem do afastamento das cúspides valvares e do consequente aumento dos refluxos venosos^14^. O retorno venoso se processa desafiando as forças da gravidade e engloba diversos mecanismos compensadores, eentre eles as bombas impulso-aspirativas (BIAs), descritas por Brizzio nos anos 1980 na Argentina. De acordo com Godoy^15^, caso um ser humano permaneça em pé e imóvel, essas bombas não atuam, e essa falha é causa associada de edema laboral^16^. No caso de indivíduos que permanecem em posição sedentária ao longo do dia de trabalho, parece ocorrer uma diminuição do trabalho musculoarticular, o que facilitaria a estase venosa.

Segundo Bishara et al.^17^, essa alteração da hemodinâmica venosa ao longo da jornada diária de trabalho pode ser consequência de alterações da competência valvular, que é fundamental para uma função venosa normal. Esses autores observaram redução significativa da capacitância venosa medida por fotopletismografia no período da tarde em relação ao período da manhã, e atribuíram essa diferença ao grande volume de sangue contido nas veias das extremidades inferiores dos indivíduos estudados depois de um período prolongado em bipedestação.

Em outro estudo, observamos que enfermeiros sem qualquer sinal de insuficiência venosa crônica que atuam em posição ortostática por 90% do tempo de trabalho apresentam altos níveis de pressão venosa dos MMII e superprodução de espécies reativas de oxigênio após o trabalho. Esses radicais livres são mediadores de danos na parede do vaso, e o dano oxidativo da membrana endotelial aumenta a permeabilidade vascular, com consequente edema^18^^,^^19^.

De acordo com Godoy^15^, 2/3 das enfermidades que acometem o sistema circulatório de retorno podem ser controladas somente pelo método de compressão. Apesar de subestimado por muitos profissionais da área de saúde, a sua eficácia vem sendo comprovada desde tempos antigos, quando hebreus e gregos usavam curativos compressivos para tratar úlceras, e soldados romanos já enfaixavam suas pernas para suportar melhor longas caminhadas durante as guerras. Hoje, com tais enfermidades ainda mais frequentes, atribui-se a alta incidência de doença venosa e linfática ao moderno estilo de vida, oriundo da industrialização e da informatização, que obriga a maioria das pessoas a permanecer em ortostatismo e em outras posições viciosas por períodos prolongados. Foi comprovado que nativos indígenas sem sinais dessas enfermidades, quando mudam para grandes centros urbanos, passam a apresentá-las^15^.

A terapia compressiva tem como objetivo principal reequilibrar os tecidos e o interstício, imprimindo uma pressão externa tal que possa contrapor-se às pressões internas intravasculares e intersticiais patológicas. A maior indicação para o seu uso é o edema^1^^,^^15^. Ela ainda age sobre os mediadores envolvidos nas reações inflamatórias localizadas em nível microcirculatório, que podem explicar o alívio sentido com a sua aplicação adequada. O aumento da velocidade microcirculatória pode ser avaliado pela fluxometria por laser-Doppler. Godoy at al.^15^ evidenciaram o aumento de oxigenação cutânea durante a compressão na insuficiência venosa. Partsch et al.^20^ demonstraram também a diminuição dos refluxos venosos, até mesmo em segmentos avalvulados.

Neste estudo, pudemos ratificar aumento volumétrico vespertino importante em ambos os MMII após jornada de 8 horas de trabalho sem uso de qualquer medida profilática contra o EO. Quanto às pacientes que não tiveram edema significativo mensurado ao final do dia, sugere-se a possibilidade de haver formação de um edema subclínico, não diagnosticado por meio da medida circunferencial. Isso poderia justificar as queixas de inchaço e peso relatadas pelas pacientes ao fim do dia. Também pode ser considerado um viés do trabalho, já que a medida circunferencial em centímetros acaba por não ser tão fidedigna e sensível a alterações menores quanto uma pletismografia de água, por exemplo. Outros estudos voltados para esse sentido são necessários para corroborar a hipótese.

Apesar de a Figura 2 demonstrar pouca diferença na circunferência da perna(apenas 1 cm de diferença entre as médias dos pontos B), pode-se inferir que os valores estatisticamente significantes foram também clinicamente significantes, pois, apesar de ainda não haver referência na literatura definindo um diâmetro exato para que o edema seja considerado importante, a correlação dos sintomas referidos pelas pacientes foi tão mais intensa e frequente quanto maiores as medidas circunferenciais.

O fato é que o EO é desconfortável, e a sensação de peso e cansaço evidenciadas pelos nossos resultados pode levar à redução da produtividade, com aumento do absenteísmo no trabalho e má qualidade de vida, e talvez seja uma das primeiras manifestações de descompensação dos sistemas venosos e linfáticos^21^, em particular em indivíduos com maiores índices de massa corporal.^22^

A maioria das participantes do estudo relatou não só uma limitação diária no trabalho devido ao problema na perna, como também limitações relacionadas às atividades diárias em sua casa (serviços domésticos, tarefas rotineiras, jardinagem, etc.). O mesmo ocorreu com as atividades sociais ou de lazer nas quais é necessário ficar de pé por longos períodos (festas, casamentos, transporte público, etc.). A melhora com o uso das MCs comprovou a sua efetividade na prevenção do EO e a sua interferência direta na qualidade de vida das cabeleireiras.

Diante disso, para a prescrição adequada de MCs para fins tanto terapêuticos quanto profiláticos, é fundamental que o médico esteja familiarizado com os valores pressóricos normais e com os valores pressóricos gerados pelas diferentes enfermidades, de modo que possa optar pela compressão que de fato combata a estase venosa, seja em condições fisiológicas ou patológicas. Segundo Partsch et al.^20^, a pressão ótima para reduzir o edema das extremidades ainda é matéria de debate. Como os indivíduos deste estudo não apresentavam aparentemente nenhuma manifestação clínica de doença venosa, optamos pela indicação de meias elásticas com níveis pressóricos de 18-20 mmHg, que se mostraram seguros e efetivos, não sendo necessária uma maior compressão. Belczak et al.^23^ concluíram em estudo recente que uma compressão de 20-30 mmHg é mais eficaz para indivíduos que trabalham na posição sentada, mas não tão significante para os que trabalham em ortostatismo. Para estes, a medida pressórica de 15-20 mmHg se manteve com bons resultados, sendo relatados benefícios a partir de 10 mmHg.

Então, além de especificar a pressão necessária em mmHg (medida universal), o médico, ao prescrever o uso de MCs, deve ainda determinar o modelo das meias (panturrilha, 7/8, meia-calça ou unilateral), indicando a marca, o período a ser utilizado e a maneira como vesti-la. As meias do tipo panturrilha potencializam a hemodinâmica venosa e são geralmente as mais indicadas, pois, além de serem fáceis de vestir e de terem uma melhor adesão do paciente, uma grande parte dos problemas flebolinfológicos se desenvolvem no terço inferior da perna. Além disso, meias confeccionadas em tecido de boa qualidade e tecnicamente corretas podem durar até 6 meses, o que representa medida acessível e econômica do ponto de vista tanto terapêutico quanto profilático^1^.

Uma limitação do estudo foi a não realização do Doppler, já que pacientes aparentemente sem doença venosa podem possuir alguma outra alteração não detectada ao exame físico. Além disso, apesar de não haver relato na literatura associando ciclo menstrual e EO, este é também um fator a ser avaliado. Sabe-se que no período pré-menstrual há uma alteração do metabolismo dos eletrólitos e da água, com retenção fisiológica de fluido extracelular (edema), resultado de uma hiperestrogenemia^24^. No nosso estudo, não foi possível correlacionar o período de menstruação com uma maior variação do edema de MMII, devido ao uso de pílulas contraceptivas por grande parte das pacientes analisadas.

Um efeito placebo poderia ser considerado um viés deste estudo. Colocar as MCs poderia, de certa forma, alterar as respostas subjetivas ao questionário de dor. Porém, consideramos que esse viés só se aplica para as pacientes cujas medidas circunferenciais não se alteraram ao final da jornada de trabalho. Afinal, para as pacientes cujo edema significativo regrediu com o uso das MCs, há uma correlação fisiológica com a melhora dos sintomas, o que valida o efeito das meias sobre o EO e seus efeitos.

Outro aspecto que deve ser ressaltado como limitação do trabalho foi a grande dificuldade na obtenção de pacientes dispostas a participar do estudo. Este foi também um fator que nos impediu de aumentar a amostra. Usar as MCs ainda é visto como algo trabalhoso e comprometedor da estética, o que dificultou a adesão ao tratamento. Esse é um problema que deve ser avaliado, já que o desfecho do tratamento é completamente dependente do uso adequado e contínuo das meias.

Fundamentados então em nossos achados e nos de outros pesquisadores já citados, recomendamos o uso das MCs como medida profilática contra o EO e suas consequências. Não ter significância estatística implica dizer que não houve diferença nas medidas de diâmetros (edema) entre a medida inicial matinal e o final do dia após usar as meias, já que todas as outras comparações entre o uso das meias e o uso sem meias foram significantes e possuíam variação em centímetros. Diante disso, é possível afirmar que as MCs preveniram o EO.

Mais do que confirmar estudos anteriores que atestaram o valor do uso de MCs como medida efetiva na prevenção do EO^19^^,^^25^^,^^26^, o fato de apresentarmos resultados significativos diante de uma amostra heterogênea, em que as participantes variaram em peso, altura e idade, deve ser visto como algo positivo, pois evidencia a efetividade das MCs na prevenção do EO.

Como o tempo de reenchimento venoso é determinado principalmente pela competência das válvulas venosas, os resultados deste estudo podem indicar que um certo grau de incompetência valvular venosa tende a se desenvolver nas extremidades inferiores normais após atividade prolongada na posição ereta. Um grau relativamente maior de incompetência valvular venosa pode ser a base fisiológica para explicar o desenvolvimento de dor ou inchaço em algumas extremidades inferiores após tais atividades.

As MCs são, portanto, uma ferramenta eficaz e econômica para a prevenção do EO e suas consequências a longo prazo, devendo o seu uso ser incentivado pelos profissionais de saúde. A venda pelas empresas responsáveis também deve ser incrementada, já que as meias de suave compressão são pouco divulgadas e podem ser vendidas sem prescrição médica, sendo ideais para esse tipo de edema.
